# Establishing a China malaria diagnosis reference laboratory network for malaria elimination

**DOI:** 10.1186/s12936-015-0556-z

**Published:** 2015-01-28

**Authors:** Jian-hai Yin, He Yan, Fang Huang, Mei Li, Hui-hui Xiao, Shui-sen Zhou, Zhi-gui Xia

**Affiliations:** National Institute of Parasitic Diseases, Chinese Center for Disease Control and Prevention, Shanghai 200025, Shanghai, People’s Republic of China; WHO Collaborating Centre for Malaria, Schistosomiasis and Filariasis, Key Laboratory of Parasite and Vector Biology, Ministry of Health, Shanghai, 200025 People’s Republic of China

**Keywords:** Malaria diagnosis, Quality assurance, Reference laboratory, Malaria elimination, China

## Abstract

**Background:**

In China, the prevalence of malaria has reduced dramatically due to the elimination programme. The continued success of the programme will depend upon the accurate diagnosis of the disease in the laboratory. The basic requirements for this are a reliable malaria diagnosis laboratory network and quality management system to support case verification and source tracking.

**Methods:**

The baseline information of provincial malaria laboratories in the China malaria diagnosis reference laboratory network was collected and analysed, and a quality-assurance activity was carried out to assess their accuracies in malaria diagnosis by microscopy using WHO standards and PCR.

**Results:**

By the end of 2013, nineteen of 24 provincial laboratories have been included in the network. In the study, a total of 168 staff were registered and there was no bias in their age, gender, education level, and position. Generally *Plasmodium* species were identified with great accuracy by microscopy and PCR. However, *Plasmodium ovale* was likely to be misdiagnosed as *Plasmodium vivax* by microscopy.

**Conclusions:**

China has established a laboratory network for primary malaria diagnosis which will cover a larger area. Currently, *Plasmodium* species can be identified fairly accurately by microscopy and PCR. However, laboratory staff need additional trainings on accurate identification of *P. ovale* microscopically and good performance of PCR operations.

## Background

**Figure 1:**
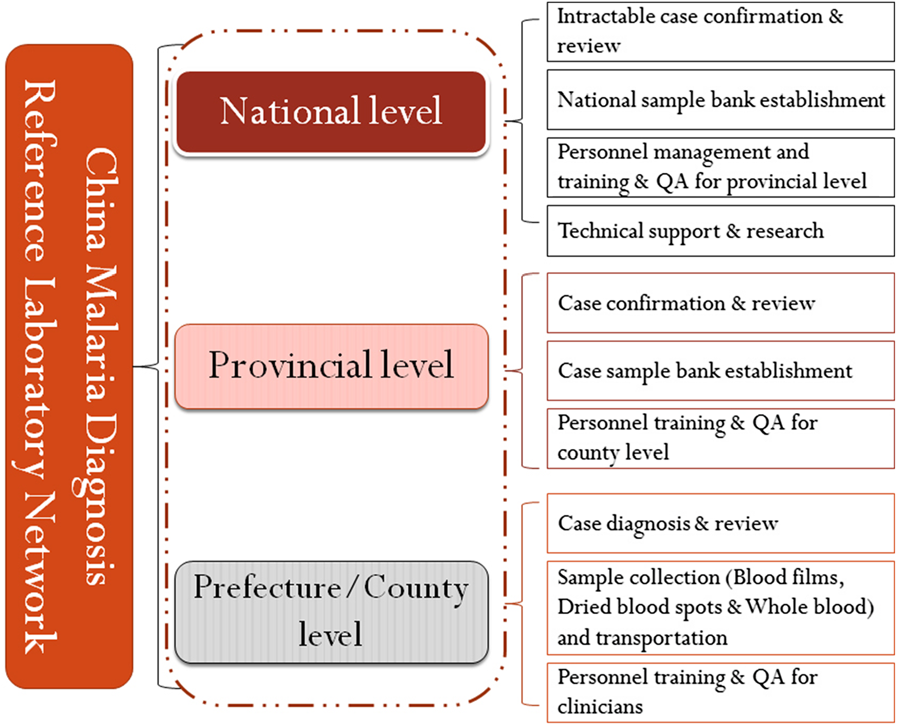
**Schematic representation of the China malaria diagnosis reference laboratory network.**

The epidemiology of malaria in China has changed dramatically [1,2], and the government embarked on a national malaria elimination programme in 2010 to eliminate malaria by 2020 in the country. One of the strategies was to establish a reliable malaria diagnosis laboratory network and a quality management system to support case verification and source-tracking. Quality assurance (QA) of malaria diagnosis was the major indicator of World Health Organization (WHO) procedures for certification of malaria elimination [3]. Consequently, it has been planned to establish the China malaria diagnosis reference laboratory network in centres for disease control and prevention or institutes of parasitic diseases at different levels mainly in the 24 historical malaria-endemic provinces [4]. Since 2011, one national and 19 provincial malaria diagnosis reference laboratories have been established. The main responsibilities of this network are case diagnosis and verification, capacity training and QA sample bank establishment, technique innovation and technical supports at different levels in order to ensure accuracy and reliability of the results (Figure 1).

The provincial and national laboratories are not only required to directly participate in the patients diagnosis and review samples from hospitals, but should also review samples diagnosed by lower level laboratories. For microscopic diagnosis each year all positive slides must be reviewed by all-level laboratories, and no less than 10% of negative slides generated in clinics, then no less than 3% of the former, and no less than 1% of the former again must be reviewed by the county prefecture and provincial levels, respectively. Finally, at least once a year the national laboratory performs reviews and QA activities to cover all the provinces.

Diagnosis in this network requires the use of light microscopy and PCR amplification of the *Plasmodium* 18s rRNA gene as mandatory methods in the provincial and national laboratories, while rapid diagnostic test (RDT) is optional. This is because using microscopy, *Plasmodium* parasites can be rapidly characterized and quantified using standard procedures [5,6], although there are some limitations in detecting asymptomatic and sub-patent infections [7,8]. In addition, the PCR assays based on 18s rRNA gene amplification have been reliable in detecting circulating parasites [9], and the nested PCR protocol to amplify this gene is widely used in laboratories, clinics and field settings [10-12].

The aim of this programme is to implement an assessment of malaria detection through microscopy and PCR in the provincial laboratories in China, and to strengthen their quality-assured capacity for malaria diagnosis.

## Methods

### Collection of baseline information

A structured questionnaire focusing on the provincial malaria laboratory was provided to each laboratory when they registered in the network.

### Participating laboratories and participants

Nineteen provincial malaria diagnosis reference laboratories were involved in the study. From each laboratory, at least two representatives participated, with one responsible for malaria diagnosis via microscopy and the other by PCR detection. They were assessed together in the national malaria diagnosis reference laboratory.

### Qualitative assessment

Qualitative assessment of microscopic observations was performed by obtaining 20 slides with Giemsa-stained malaria blood and their standard answers from the external quality assessment programme for communicable diseases (malaria microscopy) in the WHO Western Pacific Region (density unknown) (Table 1) [13]. Staff spent 10 min per slide and followed the operations in basic malaria microscopy [14] to record the results, which were not permitted to be changed after entry. For the PCR assessment, three different dried blood spots, one positive control and one negative control from the blood bank were given to each staff in a blind test for analysis. *Plasmodium* positive blood samples were previously assessed by the national malaria diagnosis reference laboratory by PCR. DNA was extracted from the blood spots using the QIA amp DNA Mini Kit (Qiagen, USA) and uniform-nested PCR was performed to amplify the multi-copy 18s rRNA gene [4,11,15]. The first PCR was performed in a 20 μl reaction volume containing 10.1 μl ddH_2_O, 1.0 μl each of rPLU5, and rPLU6 primers (10 μmol/L), 4.9 μl buffer mix, 3.0 μl DNA template in the first reaction. The nested PCR contained 11.1 μl ddH_2_O, 1.0 μl each of p1, and p2 primers (10 μmol/L), 4.9 μl buffer mix, 2.0 μl from the fisrt PCR product. The PCR conditions for both PCRs were the following: 94°C for 3 min; 34 cycles of 94°C for 30 sec, 58°C for 30 sec, and 72°C for 60 sec; and a final extension at 72°C for 5 min. All PCR supplies were from TaKaRa (Dalian, China) and primers were synthesized by Life Technologies™ (Shanghai, China). Primer sequences are provided in Table 2 [4,11,15].

**Table 1:** **Summary of the microscopic examination of malaria blood slides**

	**G1**						**G2**												**G3**
	**C**	**M**	**O**	**P**	**R**	**S**	**A**	**B**	**D**	**E**	**F**	**G**	**H**	**I**	**J**	**L**	**N**	**Q**	**K**
13-1-MM01(NE)	√	√	√	√	√	√	√	√	√	√	√	√	√	√	√	√	√	√	√
13-1-MM02(NE)	√	√	√	√	√	√	√	√	√	√	√	√	√	√	√	√	√	√	√
13-1-MM04 (*P. f*)	√	√	√	√	√	√	√	√	√	√	√	√	√	√	√	√	√	√	√
13-1-MM05 (*P. v*)	*P.o*	√	√	√	√	√	√	*P.o*	√	√	√	√	√	√	√	√	√	√	√
13-1-MM06 (*P. f*)	√	√	√	√	√	√	√	√	√	√	√	√	√	√	*P.f* + *P.v*	√	√	√	√
13-1-MM07 (*P. v*)	√	√	√	√	√	√	*P.o*	√	√	√	√	√	√	√	√	√	√	√	√
13-1-MM09 (*P. f*)	√	√	√	√	√	√	√	√	√	√	√	√	√	√	√	√	√	√	√
13-1-MM10 (*P. v*)	*P.o*	*P.o*	√	√	*P.o*	*P.o*	√	*P.o*	*P.o*	√	*P.o*	*P.o*	*P.o*	√	*P.o*	√	*P.o*	√	*P.o*
13-1-MM14 (NE)	√	√	√	√	√	√	√	√	√	√	√	√	√	√	√	√	√	√	√
13-1-MM15 (*P. m*)	√	√	√	√	√	√	√	√	√	√	√	√	√	√	√	*P.v*	√	√	√
13-2-MM01(*P. f*)	√	√	√	√	√	√	√	√	√	√	√	√	√	√	√	√	√	√	√
13-2-MM02(NE)	√	√	√	√	√	√	√	√	√	√	√	√	√	√	√	√	√	√	√
13-2-MM03 (*P. f*)	√	√	√	√	√	√	√	√	√	√	√	√	√	√	√	√	√	√	√
13-2-MM04 (*P. v*)	√	*P.f*	√	√	√	√	√	√	√	P.m	√	√	√	√	√	√	√	√	√
13-2-MM05 (*P. o*)	*P.m*	√	√	√	√	*P.v*	√	√	*P.v*	√	√	*P.v*	√	*P.v*	*P.m*	*P.v*	√	*P.k*	*P.v*
13-2-MM06 (*P. f*)	√	√	√	√	√	√	√	√	√	√	√	√	√	NE	√	√	√	√	√
13-2-MM08 (NE)	√	√	√	√	√	√	√	√	√	√	√	√	√	√	*P.v*	√	√	√	*P.v*
13-2-MM10 (*P. v*)	*P.o*	√	√	√	√	√	√	√	√	√	√	√	√	√	*P.o*	√	√	√	√
13-2-MM14 (*P. v*)	√	√	√	√	√	√	√	*P.o*	√	√	√	√	√	√	*P.o*	√	√	√	√
13-2-MM15 (*P. f*)	√	√	√	√	√	√	√	√	√	√	√	√	√	√	√	√	√	√	√

√ indicates correct identification; NE indicates that the slide is negative for *Plasmodium* spp; different alphabets in the title row indicate the different staff in participating laboratories.

**Table 2:** **PCR primers used and expected product size in malaria diagnosis**

**Reaction**	**Primer name**	**Primer sequence**	**Product length**
First PCR	rPLU5	**5′**-CCTGTTGTTGCCTTAAACTTC-**3′**	1200 bp
	rPLU6	**5′**-TTAAAATTGTTGCAGTTAAAACG-**3′**	
Nested PCR	rFAL1	**5′**-TTAAACTGGTTTGGGAAAACCAAATATATT-**3′**	205 bp
	rFAL2	**5′**-ACACAATGAACTCAATCATGACTACCCGTC-**3′**	
	rVIV1	**5′**-CGCTTCTAGCTTAATCCACATAACTGATAC-**3′**	120 bp
	rVIV2	**5′**-ACTTCCAAGCCGAAGCAAAGAAAGTCCTTA-**3′**	
	rMAL1	**5′**-ATAACATAGTTGTACGTTAAGAATAACCGC-**3′**	141 bp
	rMAL2	**5′**-AAAATTCCCATGCATAAAAAATTATACAAA-**3′**	
	rOVA1	**5′**-ATCTCTTTTGCTATTTTTTAGTATTGGAGA-**3′**	800 bp
	rOVA2	**5′**-GGAAAAGGACACATTAATTGTATCCTAGTG-**3′**	

### Data analysis

Baseline information from each laboratory was verified by an expert group (two malaria experts from Subcommittee for Schistosomiasis and Parasitic Diseases, Expert Advisory Committee for Disease Control, Ministry of Health, PR China, and two from national malaria diagnosis reference laboratory) through field investigation. All collected data were entered into Microsoft Excel and analysed using SAS 9.2. A *P* value <0.05 was considered statistically significant.

### Ethical clearance

The programme was reviewed and approved by the Ethical Committee of National Institute of Parasitic Diseases, China CDC. All participants provided written informed consent for the study.

## Results

**Figure 2:**
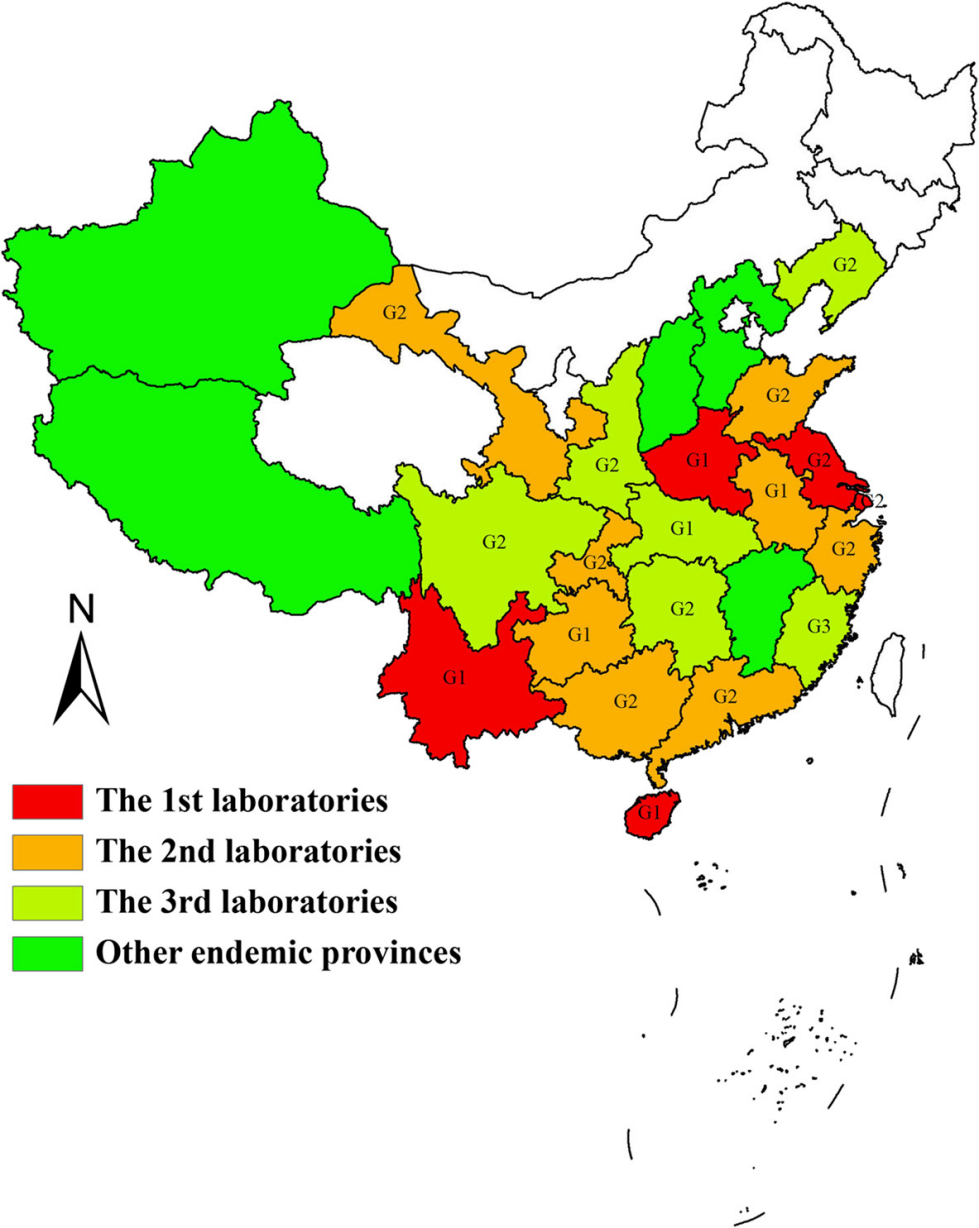
**Distribution and classification of the provincial malaria diagnosis reference laboratories.**

In total, 19 provincial reference laboratories that met all requirement for inclusion in the network such as laboratory room, devices, organization, and technical demands were established in 2011, 2012 and 2013, respectively (Figure 2). All these laboratories had independent rooms for the following: sample storage, microscopy and molecular biological detection (PCR), and essential devices, such as microscopes. They also had normal and low or ultra-low temperature refrigerators, water baths, autoclaves, high-speed centrifuges, DNA amplification system, electrophoretic imaging system.

### Laboratory staff

A total of 168 staff from 19 provincial malaria laboratories registered in the programme. Each laboratory had –four to 14 individuals (8.84 ± 2.87 persons/laboratory) ranging in ages from 25 - 59 years (40.26 ± 10.62) with 61.9% being males (104/168) and 38.1% females (64/168). Among them 45.8% (77/168) had bachelors’ degree and 35.7% (60/168) had a graduate degree and the remaining had a college degree. In addition, 96 individuals (5.05/laboratory) were full-time staff of the malaria laboratory while 72 worked part-time. The position of the individuals was responsible for laboratory management, microscopy, molecular biological detection, and management of QA and biosafety, respectively.

The 19 laboratories were classified into three groups (G1 ~ 3) (Figure 2) based on the geographical malaria epidemiology area described in the Action Plan of Malaria Elimination (2010-2020) and a previous publication [16]. These demographics among groups were not statistically significant. Age: Kruskal-Wallis Test, χ^2^ = 0.1539, *P* = 0.9259. Gender: Fisher’s exact probability, *P* = 0.7677. Education level: Fisher’s exact probability, *P* = 0.3238. Work style (full-time or part-time): Fisher’s exact probability, *P* = 0.6412.

### Microscopy assessment

In total, only two participants correctly identified all 20 slides. Seven failed in one slide, six incorrectly identified two slides, two individuals failed in three slides, and one each incorrectly identified four and six slides, respectively. Particularly, 12 participants identified the *Plasmodium vivax*-positive slide marked ‘13-1-MM10’ as *Plasmodium ovale*. The *P. ovale* positive slide of ‘13-2-MM05’ was the most difficult for participants with nine failing to identify it and most of them (6/9) incorrectly identifying it as *P. vivax* (Table 1). However, no significant differences in error percentages were observed among the three groups (Fisher’s exact probability, *P* = 0.2338).

### PCR assessment

Among the results reported, 84.21% (48/57) of samples were detected correctly; 63.16% (12/19) of staff detected all samples correctly and 26.32% (5/19) failed in one sample. Interestingly, two participants detected only one sample correctly (Table 3). No significant differences were found among the three groups (Fisher’s exact probability, *P* = 0.3569).

**Table 3:** **Malaria diagnosis by PCR**

	**G1**						**G2**												**G3**
	**C1**	**M1**	**O1**	**P1**	**R1**	**S1**	**A1**	**B1**	**D1**	**E1**	**F1**	**G1**	**H1**	**I1**	**J1**	**L1**	**N1**	**Q1**	**K1**
Correct diagnosis	2	2	3	2	1	2	3	3	2	1	3	3	3	3	3	3	3	3	3
Wrong diagnosis	1	1	0	1	2	1	0	0	1	2	0	0	0	0	0	0	0	0	0

Different alphabets in the title row indicate the different staff in participating laboratories.

## Discussion

This is the first major diagnosis quality assessment in China malaria diagnosis reference laboratory network, and such activities are critical for the reduction or elimination of the disease in the country. As there are 5.5 persons on average in each laboratory the participants in the study can be seen as representative of capacity of their own laboratory, even though not randomly drawn. The results reported here provide insights into the efficacy of malaria diagnosis using microscopy and PCR methods, and highlight areas that require improvement.

Currently, participants in the provincial laboratories can efficiently identify most *Plasmodium* species by light microscopy with an accuracy level of 90.79% (345/380). Besides, microscopists (O, P and Q), who achieved the top level 1, 2 or 3 efficiency in diagnosing malaria through a microscope and were assessed in China by a WHO-Certified Level-1/expert microscopist in 2012 [17], in the present programme maintained high efficiency levels. Moreover, in the three external quality assessment programmes (EQAPs) for malaria microscopy (2012-2013) organized by WHO, the accuracy of identifying *Plasmodium* species by microscopists in national laboratory was 91.67 (55/60), 86.67 (39/45) and 95.56% (43/45). Overall, these demonstrated the high competency of *Plasmodium* species identification by Chinese malaria microscopists.

However, malaria diagnosis in China still faces the challenges. First, the microscopy competency at lower levels needs improvement. The average accuracy of *Plasmodium* detection in China ranges from 62 to 66%, which is based on three national technique competitions for diagnosis of parasitic diseases conducted in 2011 [18], 2012 [16] and 2013 (unpublished). In these competitions, the accuracy of *Plasmodium falciparum* and *P. vivax* species identification was about 65% [16,18]. In each competition, participants from below provincial levels had the lower scores. Since a positive case of malaria in an elimination programme is defined as the presence of parasites in the blood regardless of the presence or absence of clinical symptoms [4,19,20], to improve the basic capability for malaria diagnosis, it is essential to equip existing staff with reliable identification techniques.

Second, it is difficult to identify rare *Plasmodium* species in China. Although total malaria cases reported in recent years decreased markedly, malaria importation poses a constant threat to mainland China with the proportion increasing every year. The major species imported to China are the dominant *P. falciparum* from African continent and *P. vivax* from Asian countries, but other species, such as *Plasmodium malariae* and *P. ovale* have also been reported [1,21,22].

*Plasmodium ovale* has been reported throughout the world, and its natural distribution [23] is limited to sub-Saharan Africa [24-27] and the islands of the western Pacific [28-30]. In China, *P. ovale* received little attention primarily due to sporadic malaria cases associated with this species [31]. The incidence of *P. ovale* malaria has increased due to importation by travellers to sub-Saharan Africa [32-36]. The symptoms of *P. ovale* malaria include common cold-like mild symptoms and are accompanied with low parasitaemia, and individuals infected this species are given the same treatment as *P. vivax* [37]. It has been reported previously that it is difficult to differentiate *P. ovale* from *P. vivax* by examining peripheral blood films stained with Giemsa [37]. Consistent with this report, the *P. ovale* malaria cases in China were misdiagnosed as *P. vivax* malaria [38,39] as was the case in this present programme. Therefore, for the successful elimination of malaria in China, it is essential to pay attention to the identification of *P. ovale*, and strengthen the training at different levels.

The diagnosis of malaria parasites by nested PCR in the present assessment is at medium level with an accuracy of 84.21% (48/57). The ease and speed of PCR makes it an important tool in malaria elimination programmes and should be improved to detect asymptomatic infections. Since various PCR methods have been developed for malaria diagnosis [40-44], it is necessary to compare the benefits of different methods, optimize the protocol, and train staff in the reference laboratory network. Meanwhile, more samples with different densities should be included in future QA activities.

In addition, more comprehensive assessments including slides and dry blood spots with different parasite densities should be considered in future.

## Conclusion

A malaria diagnosis reference laboratory network has been established in China and is in its initial stages. The identification of *Plasmodium* species using microscopy and PCR is at medium to high level. The biggest problem is the misdiagnosis of *P. ovale* as *P. vivax* microscopically. It is essential for more laboratories at different levels apply to join the network to obtain the required training and sustain malaria elimination in China. Special emphasis should be on the identification of locally rare *Plasmodium* species, such as *P. ovale*.
